# Endoscopic characteristics and clinical outcomes of squamous intraepithelial lesions and squamous cell carcinoma in the anal canal

**DOI:** 10.1055/a-2816-5093

**Published:** 2026-04-17

**Authors:** Takeshi Uozumi, Yasuhiko Mizuguchi, Ryoji Ichijima, Naoya Toyoshima, Masau Sekiguchi, Hiroyuki Takamaru, Masayoshi Yamada, Nozomu Kobayashi, Taiki Hashimoto, Yutaka Saito

**Affiliations:** 1 Endoscopy Division, National Cancer Center Hospital, Tokyo, Japan; 2 Cancer Screening Center, National Cancer Center Hospital, Tokyo, Japan; 3 13543 Division of Molecular Modification and Cancer Biology, National Cancer Center Research Institute, Tokyo, Japan; 4 Division of Diagnostic Pathology, National Cancer Center Hospital, Tokyo, Japan

**Keywords:** Endoscopy Lower GI Tract, Colorectal cancer, Diagnosis and imaging (inc. chromoendoscopy, NBI, iSCAN, FICE, CLE...), Endoscopic resection (polypectomy, ESD, EMRc, ...)

## Abstract

**Background and study aims:**

Squamous epithelial lesions, including low- and high-grade squamous intraepithelial lesions (LSILs and HSILs) and anal squamous cell carcinoma (ASCC), are rare diseases. Consequently, few reports have precisely characterized their endoscopic features or reported clinical outcomes following endoscopic submucosal dissection (ESD). In this case series, we describe detailed endoscopic characteristics and clinical outcomes of squamous epithelial lesions.

**Patients and methods:**

This was a single-center retrospective case series. Data from a consecutive series of patients who underwent ESD for squamous epithelial lesions between 2014 and 2024 were retrieved from the integrated database. Two expert endoscopists reviewed endoscopy images. Diagnostic performance of the Japan Esophageal Society (JES) classification was also investigated.

**Results:**

A total of 18 lesions from 11 patients were identified. Diagnoses after ESD were as follows: LSIL in three lesions, HSIL in 12 lesions, and ASCC in three lesions. LSILs appeared as slightly elevated lesions with whitish, papillomatous surfaces and exhibited Type A vessels. HSILs were flat or slightly elevated and showed Type B1 vessels. ASCCs presented with reddish or protruding components and exhibited Type B2 or B3 vessels at the regions. Diagnostic accuracy of JES classification was 92% (11/12; 95% confidence interval 65–99). En bloc and complete resection rates were 100% and 56%, respectively. There was no local recurrence or distant metastasis with median follow-up 12 months.

**Conclusions:**

Anal squamous epithelial lesions demonstrated characteristic findings. JES classification using magnifying narrow-band imaging might be useful, although limitations remain in detecting flat HSILs in the anal transitional zone.

## Introduction

Anal squamous cell carcinoma (ASCC) is a rare malignancy with a prevalence of 1.6 per 100,000 person-years in the general populations 1 . However, incidence and mortality of ASCC have been increasing during the last several decades 1 . ASCC is caused by human papillomavirus (HPV) infection and is preceded by a squamous intraepithelial lesion (SIL) 2 . The Lower Anogenital Squamous Terminology (LAST) project proposed a dichotomous terminology for SIL: low-grade SIL (LSIL) and high-grade squamous SIL (HSIL) 3 . LSIL is a transient lesion caused by productive HPV infection 4 . From a clinical perspective, LSIL sometimes regresses spontaneously and is considered to have a limited risk of malignant transformation. By contrast, HSIL is a persistent lesion caused by proliferative oncogenic HPV infection 4 . HSIL is a precursor of ASCC and treatment of it decreases approximately 60% of ASCC incidence 5 . Considering the risk of malignant transformation, LSIL can be treated conservatively, although HSIL requires local treatment, including endoscopic resection or ablation. Chemoradiotherapy is the current standard treatment for ASCC; however, superficially invasive ASCC is potentially amenable to local resection 6 . Accordingly, treatment strategy for squamous epithelial lesion (SEL) varies depending on the pretreatment diagnosis, highlighting the clinical significance of accurate pretreatment assessment.

Accurate endoscopic diagnosis of SELs in the anal canal is particularly challenging owing to their rarity and the unique anatomical characteristics of the anal canal. Because the endoscopic diagnosis of SELs is not well established, biopsy remains the gold standard for preoperative diagnosis. Nevertheless, biopsy represent limited samples within fields of disease, leading to underrepresentation of the actual disease. Such underestimation might lead to inappropriate treatment, potentially resulting in disease progression and a missed opportunity for curative treatment. Although obtaining multiple biopsies from different areas might help reduce sampling error, repetitive biopsies cause fibrosis, making subsequent endoscopic resection challenging. Therefore, there is a critical need to establish endoscopic diagnostic criteria for SELs to improve preoperative diagnostic accuracy.

Although several studies have reported the application of the magnifying endoscopy for SELs of the anal canal, an exclusive focus on magnified findings may provide an incomplete understanding of these lesions 7 8 . Non-magnified endoscopic findings represent the first step in lesion recognition during routine endoscopy and play a pivotal role in determining whether targeted biopsy or therapeutic intervention is warranted. However, detailed descriptions of endoscopic features based on non-magnified observation remain scarce. Therefore, the aim of this study was to precisely describe the endoscopic characteristics of SELs of the anal canal, including non-magnified white-light imaging. In addition, we investigated the clinical outcomes and safety of endoscopic resection as a local treatment for superficial SELs.

## Patients and methods

### Patients

A consecutive series of patients preoperatively diagnosed with SELs by endoscopic findings and/or biopsy at the National Cancer Center Hospital between January 2014 and August 2024, were retrieved from the integrated database of endoscopy and pathology reports 9 . Among these patients, patients with histologically confirmed diagnoses based on endoscopically resected specimens were included in the present study. Patients who were managed with biopsy alone were excluded because biopsy alone may not provide an accurate diagnosis. Patients with advanced cancer or those who received chemoradiotherapy or surgical treatment as a first-line treatment were also excluded. The medical records of the patients were reviewed to collect their clinical characteristics, including age, sex, history of HIV infection. This study was approved by the Ethics Committee of the National Cancer Center, Tokyo, Japan, and was conducted in accordance with the Declaration of Helsinki.

### Treatment strategy for SELs in the anal canal

There is no standardized treatment guideline for superficial SELs in Japan; therefore, treatment strategy was discussed for each patient at the endoscopy division conference or multidisciplinary team. Considering limited reproducibility of biopsy and risk of underrepresentation of actual disease, we principally selected endoscopic submucosal dissection (ESD) as a local treatment for superficial lesions that were diagnosed as LSIL, HSIL (including squamous cell carcinoma in situ), or superficially invasive ASCC by biopsy.

Image-enhanced endoscopy, including narrow band imaging (NBI)/blue laser imaging and chromoendoscopy (indigo carmine or Lugol staining) were used as needed for detailed examination, and ESD was performed as previously described 10 . Follow-up or additional treatment was decided by attending physician considering the patient’s age, comorbidities, and pathological results following ESD.

### Definition of endoscopic findings

The macroscopic type was assessed according to the Paris endoscopic classification and divided into two groups: protruding (type 0-Ip or 0-Is) and non-protruding (type 0-IIa, 0-IIb or 0-IIc) 11 12 . Tumor coloration was assessed based on the predominant color tone and was categorized into two groups: reddish or isochromatic/whitish.

Previous case reports suggested that vessel structures assessed by the Japan Esophageal Society (JES) magnifying endoscopy classification might reflect the histological severity and invasion depth; therefore, we evaluated the vessel structures with magnifying NBI (M-NBI) using the JES classification 8 13 14 15 . According to the JES classification, the vessel irregularity was evaluated for the following morphological factors: tortuosity, dilation, irregular caliber, and various shapes. Vessel structures were classified as Type A if they did not meet all four morphological factors and Type B if they had all morphological factors. Type B was subclassified into B1, B2 and B3 based on the running patter or degree of dilation of severely irregular vessels ( **Supplementary Table 1** ).

Two expert endoscopists (TU and NT), board-certified fellows of the Japanese Gastroenterological Endoscopy Society, reviewed the stored endoscopic images and reports. In case of discordance in the endoscopic findings among the endoscopists, consensus was reached through discussion.

### Clinical outcome assessment

Local recurrence following ESD was assessed by surveillance colonoscopy. Biopsy specimens were obtained when local recurrence was suspected. In patients with ASCC, local recurrence and distant metastasis were additionally evaluated using contrast-enhanced computed tomography. Recurrence was defined as either histologically confirmed recurrence or clinically evident recurrence based on endoscopic or radiologic findings. These clinical outcomes were assessed as on February 28, 2025.

### Histological diagnosis

LSIL, HSIL and ASCC were diagnosed according to the LAST project statement and WHO classification 3 16 . LSIL shows cytological atypia and mitotic figures in the lower third of the epithelium, and/or is characterized by koilocytosis with perinuclear haloes. LSIL is consistent with the lesions that are previously classified as anal squamous intraepithelial lesion (AIN) 1 or condyloma acuminatum 3 . HSIL shows involvement of two-thirds or more of the squamous epithelium by marked cytological atypia, mitotic figures in the upper two-thirds of the epithelium and loss of nuclear polarity. HSIL is consistent with lesions that are previously classified as AIN 2, AIN 3, or SCC in situ 3 . ASCC was defined as invasive SCC and invasion depth was measured from the surface of the tumor to the deepest point of the lesion 3 . Strong and diffuse block staining for p16 is diagnosed as p16-positive 3 . Histopathological diagnoses were confirmed by a pathologist (TH) specializing in gastrointestinal pathology.

### Diagnostic accuracy of M-NBI for SIL

We evaluated diagnostic accuracy of M-NBI using JES classification 13 . In this analysis, based on the previous reports, we hypothesized the relationships between endoscopic finding and pathological result as follows: Type A-LSIL, Type B1-HSIL, and Type B2 or B3-ASCC 8 13 14 15 17 . Images focusing on the region of interest using M-NBI were evaluated in this analysis. Lesions that were not detected or underwent detailed examination with M-NBI at the preoperative endoscopy were excluded from this analysis.

### Statistical analysis

Continuous variables were summarized using median with ranges. Diagnostic accuracy, sensitivity, specificity, positive predictive value (PPV), and negative predictive value (NPV) were calculated for each M-NBI finding. These values were demonstrated as proportion with 95% confidence interval (CI). The CI was calculated using Wilson’s score method. All statistical analyses were performed using R version 4.3.3 (R Foundation for Statistical Computing, Vienna, Austria).

## Results

### Clinical features of patients

A total of 65 patients were diagnosed with SELs by endoscopic findings and/or biopsy between January 2014 and August 2024. Patients who underwent chemoradiotherapy (47 patients), surgical resection (2 patients), systemic chemotherapy (1 patients), or follow-up with biopsy alone (4 patients) were excluded. Consequently, 11 patients with 18 lesions were included in this analysis (two patients had multiple lesions), comprising three lesions of LSIL, 12 lesions of HSIL, and three lesions of ASCC. Median age was 63 years (range, 31–75 years), and approximately half of the patients were male (55% [6/11]). Information on sexual behavior and HPV vaccination were not available in the medical records. The majority of patients were asymptomatic and underwent colonoscopy for screening (fecal occult blood test positive or primary colorectal cancer screening) or surveillance after endoscopic resection for adenomas. All patients were HIV negative ( Table 1 ).

**Table:** **Table 1** Characteristics of the 11 patients and ESD results.

Patients		n = 11
Age (years)	median [range]	63 [31–75]
Sex	Male	6
	Female	5
Lesions		n = 18
Procedure time (min)	median [range]	112 [20–160]
Lesion size (mm)	median [range]	10 [1–70]
Resected specimen size (mm)	median [range]	44 [23–80]
En-bloc (%)		100 (18/18)
Complete resection (%)		56 (10/18)
Adverse events		
Delayed bleeding, n (%)		2 (18)
Stricture, n (%)		1 (9)
En-bloc resection was defined as removal of lesions in a single piece without fragmentation. Complete resection was defined as histologically negative for vertical and horizontal margin. ESD, endoscopic submucosal dissection.		

### Endoscopic characteristics of SELs

#### LSIL

**Fig. 1:**
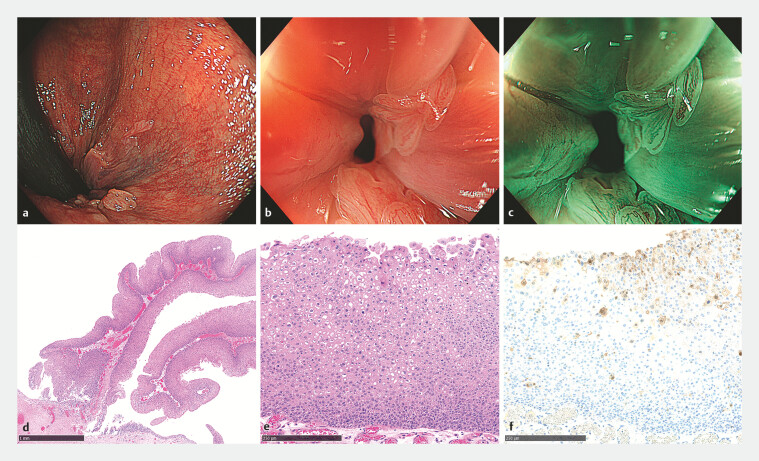
Endoscopic findings and histopathological characteristics of low-grade squamous intraepithelial lesion. **a** Papillomatous slightly elevated lesions were located in the anal canal. **b** Forward view. **c** Magnifying NBI revealed irregular vessels with tortuosity, dilation, and various shapes. The caliber was regular. These vessels were diagnosed as Type A vessels (bobby pin-like shape). **d** Dilated and tortuous vessels were detected in the papillomatous configuration (black bar was 500 μm). **e** Cytological atypia in the lower third of the epithelium and koilocytosis with perinuclear haloes were observed (black bar was 250 μm). **f** p16 staining was negative (black bar was 500 μm).

LSIL was detected in three lesions from two patients. These were 5, 7, and 60 mm in size, and the largest one occupied the semi-circumference of the anal canal. The macroscopic type of them were non-protruding (0-IIa) and characterized by whitish papillomatous structures. M-NBI showed Type A vessels (bobby pin-like shape) in two of three lesions and Type B1 vessels in the other lesion ( Fig. 1 , Table 2 ).

**Table:** **Table 2** Endoscopic characteristics of squamous epithelial lesions.

		LSIL n = 3	HSIL n = 12	ASCC n = 3
Size (mm)	Median [range]	7 [5–60]	5 [1–70]	50 [24–53]
Color (n)	Whitish/isochromatic	3	7*	2
	Reddish	0	0	1
Macroscopic type (n)	Protruding	0	0	2
	Non-protruding	3	12	1
JES classification	Type A (n)	2	0	0
	Type B1 (n)	1	6 ^†^	0
	Type B2 or 3 (n)	0	0	3
*Five HSILs were not endoscopically recognized prior to resection and were subsequently diagnosed based on histological evaluation. ^†^ The other cases were not investigated with magnifying narrow band imaging. ASCC, anal squamous cell carcinoma; HSIL, high-grade squamous intraepithelial lesion; JES classification, Japan Esophageal Society classification; LSIL, low-grade squamous intraepithelial lesion.				

#### HSIL

**Fig. 2:**
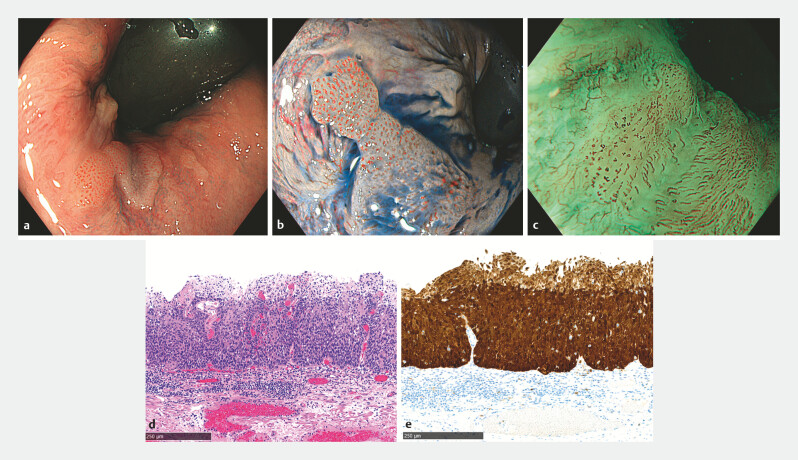
Endoscopic findings and histopathological characteristics of high-grade squamous intraepithelial lesion. **a** end pale color, slightly elevated lesion with smooth surface was located in the anal canal. **b** Texture and color enhancement imaging (TXI) with indigo carmine spraying highlight the boundary. **c** M-NBI revealed dot-shaped irregular vessels with tortuosity, dilation, irregular caliber, and various shapes. These vessels were diagnosed as Type B1 (dot-like shape). **d** Marked cytological atypia was observed in the entire thickness of the epithelium (black bar was 250 μm). **e** p16 staining was diffusely positive (black bar was 250 μm).

HSIL was detected in 12 lesions from seven patients. Median size was 5 mm (range: 1–70 mm) and the macroscopic type was non-protruding in all cases (0-IIa in seven lesions and 0-IIb in five lesions). Among the 12 lesions, five were not detected endoscopically before ESD and were only identified histologically in the resected specimens. All five lesions that were undetected at preoperative endoscopy were flat and less than 5 mm in size. Coloration of the seven lesions that were recognized at preoperative endoscopy was whitish or isochromatic. M-NBI was performed for six lesions, excluding the five endoscopically undetected lesions and one lesion in which M-NBI was not performed, and the six lesions showed Type B1 vessels (dot-like shape) in all lesions ( Fig. 2 , Table 2 ).

#### ASCC

**Fig. 3:**
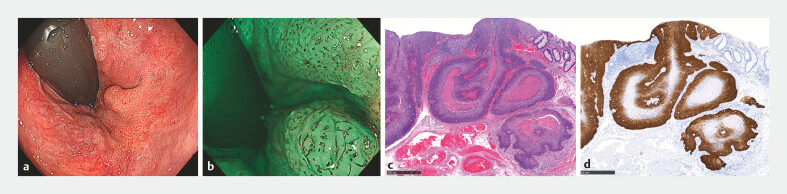
Endoscopic findings and histopathological characteristics of anal squamous cell carcinoma. **a** A slightly elevated lesion was located in the anal canal. A large nodule was detected in the middle. **b** M-NBI revealed irregular vessels without loop structure in the nodule. These vessels were diagnosed as Type B2. **c** Invasive squamous cell carcinoma. Dilated irregular vessels were observed (Black bar was 500 μm). **d** Diffuse expression of p16 (black bar was 500 μm).

ASCC was detected in three lesions from three patients. These were 24, 50, and 53 mm in size and macroscopic type was non-protruding (0-Is) in one lesion and protruding (0-IIc) in two lesions. Coloration was reddish in one lesion and isochromatic in two lesions. M-NBI showed Type B2 vessels at region of interest (reddish area or protruding component) in all lesions ( Fig. 3 , Table 2 ).

### Diagnostic accuracy of JES classification for SIL

Among the 18 lesions, six were excluded from the M-NBI analysis, including five undetected HSILs and one lesion that did not undergo detailed preoperative M-NBI examination. Thus, 12 lesions were evaluable for diagnostic accuracy analysis.

Diagnostic accuracy of JES classification was 92% (95% CI 65–99). The sensitivity, specificity, PPV, and NPV for Type A vessels with M-NBI to predict LSIL were 67%, 100%, 100%, and 90%, respectively. Sensitivity, specificity, PPV, and NPV for Type B1 vessels with M-NBI to predict HSIL were 100%, 83%, 86%, and 100%, respectively. Sensitivity, specificity, PPV, and NPV for Type B2 or 3 vessels with M-NBI to predict ASCC were 100%, 100%, 100%, and 100%, respectively ( Table 3 ).

**Table:** **Table 3** Diagnostic accuracy of JES classification and biopsy.

	Sensitivity	Specificity	PPV	NPV	Accuracy
JES classification, % (n, [95% CI])					92 (11/12 [95% CI 65–99])
Type A for LSIL, % (n, [95%CI])	67 (2/3 [95% CI 20–94])	100 (9/9 [95% CI 70–100])	100 (2/2 [95% CI 34–100])	90 (9/10 [95% CI 60–98])	
Type B1 for HSIL, % (n, [95% CI])	100 (6/6 [95% CI 61–100])	83 (5/6 [95% CI 44–97])	86 (6/7 [95% CI 49–97])	100 (5/5 [95% CI 57–100])	
Type B2 or 3 for ASCC, % (n, [95% CI])	100 (3/3 [95% CI 44–100])	100 (9/9 [95% CI 70–100])	100 (3/3 [95% CI 44–100])	100 (9/9 [95% CI 70–100])	
ASCC, anal squamous cell carcinoma; CI, confidence interval; HSIL, high-grade squamous intraepithelial lesion; JES classification, Japan Esophageal Society classification; LSIL, low-grade squamous intraepithelial lesion; NPV, negative predictive value; PPV, positive predictive value.					

### ESD for SILs

Median procedure time was 112 minutes (range, 20–160 minutes). Median lesion size and resected specimen size were 10 mm (range, 1–70 mm) and 44 mm (range, 23–80 mm), respectively.

En bloc and complete resection rates were 100% (18/18) and 56% (10/18), respectively. Among the eight cases of incomplete resection, the relatively low R0 resection rate was mainly attributable to pHMX at the anal side (6/8); the remaining cases included one pHM1 and one pVM1. Median invasion depth of ASCC was 3000 μm (range, 2000–3750 μm), and all lesions were lymphovascular invasion positive ( Table 1 ).

### Short-term and long-term outcomes of squamous epithelial lesions following ESD

Delayed bleeding and stricture were observed in 18% (2/11) and 9% (1/11) of cases, respectively. The stricture was eliminated after a single session of endoscopic balloon dilation. All patients diagnosed with ASCC underwent chemoradiotherapy following ESD, whereas those diagnosed with LSIL or HSIL were managed with surveillance without additional treatment. Median follow-up period was 12 months (range, 6–63 months), and there was no local recurrence or distant metastasis ( Table 1 ).

## Discussion

In this study, we investigated endoscopic characteristics of SELs in the anal canal. LSILs appeared as slightly elevated (0-IIa) lesions with whitish, papillomatous structure and exhibited Type A vessels (bobby pin-like shape) with M-NBI. HSILs were flat (0-IIb) or slightly elevated (0-IIa) and showed Type B1 vessels (dot-like shape). ASCCs presented with reddish or protruding components (0-Is) and exhibited Type B2 vessels at these regions. We believe these findings will help to improving endoscopic diagnostic accuracy.

Although the aforementioned endoscopic features of anal canal SELs were identified, it should be noted that five HSILs were not detected endoscopically before ESD and were only identified histologically in the resected specimens. This finding highlights an important limitation of endoscopic diagnosis in the anal canal. These undetected HSILs were small (< 5 mm) and completely flat (0-IIb) and were located in the transitional epithelium. The oral side of the anal canal is lined by transitional epithelium, which is characterized by a mixture of squamous and columnar epithelium. Moreover, the anal canal mucosa is frequently affected by background inflammatory changes or hemorrhoidal disease. These complex histological backgrounds may obscure demarcation lines between neoplastic and non-neoplastic mucosa, particularly in small lesions. Therefore, when a SEL is suspected in the anal canal, especially near the transitional zone, endoscopists should be aware of the possibility of coexisting HSILs that are not endoscopically apparent. Given the difficulty of delineating accurate demarcation lines, endoscopic resection involving anal transitional zone might help overcome this issue 18 . Careful pathological evaluation of resected specimens and post-procedural surveillance are essential to compensate for this diagnostic limitation.

Diagnostic accuracy of the JES classification for SELs was favorable, and no underestimation occurred when Type A was diagnosed as LSIL, Type B1 as HSIL, and Type B2 or B3 as ASCC. JES classification appears to be applicable for diagnosis of SELs in the anal canal; however, further large-scale studies are warranted to validate the utility of M-NBI for SELs in the anal canal.

Standardized endoscopic diagnostic criteria and treatment strategies for SELs, especially for ASCC, have not been established in the current Japanese guidelines 19 . Therefore, ESD was primarily performed as a diagnostic staging method to obtain an en bloc specimen for accurate histopathological diagnosis, whereas curative resection was also intended whenever feasible. As a result, chemoradiotherapy was selectively administered only to patients with ASCC, whereas those with LSIL or HSIL were managed with local resection alone, avoiding overtreatment. Although the complete resection rate was relatively low, this was mainly attributable to intentional limitation of the resection margin on the anal side to prevent unnecessary perianal skin resection. Despite incomplete resection in some cases, no local recurrence was observed during follow-up, and no additional treatment was required for margin-positive cases. Nevertheless, we acknowledge that careful and continued endoscopic follow-up is essential in such cases.

In our study, post-ESD stricture occurred in one patient who underwent circumferential resection involving the transitional epithelium. Given the potential risk of refractory stricture after extensive endoscopic resection and possibility of delaying subsequent chemoradiotherapy, ESD should be performed with particular caution in circumferential or semi-circumferential lesions, especially those exhibiting B2 or B3 vessels. In such cases, chemoradiotherapy may be more appropriate as the initial treatment. Overall, ESD-based management may be feasible for selected anal canal SEL.

This study had several limitations. First, this was a single-center retrospective study with a small sample size, which limits generalizability of our findings. Second, HPV status was not evaluated in this study, although it is known to be associated with risk of malignant transformation. Third, this study did not investigate SELs that were not treated with endoscopic resection, which may have introduced selection bias. Fourth, the follow-up period was relatively short to evaluate the effectiveness of adjuvant chemoradiotherapy following ESD for ASCC. Although chemoradiotherapy is a standard treatment for ASCC, clinical significance of adjuvant chemoradiotherapy after ESD has not been established. Therefore, further large-scale studies are warranted to evaluate it. Finally, the role of endoscopic resection for LSIL remains controversial. LSIL is believed to be a transient lesion that spontaneously regresses without intervention. However, in one patient in our case series, LSIL and HSIL unexpectedly coexisted, presumably due to a mixed infection with low- and high-risk HPV. In this patient, HSIL was not detected at the preoperative endoscopy and was incidentally resected along with LSIL by ESD. This finding highlights the potential limitation of preoperative biopsy and underscores the importance of careful surveillance. Although our data are insufficient to support routine endoscopic resection for LSIL, diagnostic ESD with careful informed consent regarding this potential risk might be considered as an option in selected cases.

## Conclusions

In conclusion, several cases showed multiple lesions, coexistence of LSIL and HSIL, or undetectable HSILs. JES classification using M-NBI might be useful, although limitations remain in detecting flat HSILs on anal transitional zone.

## Supplementary Material

Supplementary Material Zusatzmaterial
