# Supplementary material for: Endoscopic characteristics and clinical outcomes of squamous intraepithelial lesions and squamous cell carcinoma in the anal canal
Source: Endosc Int Open. 2026 Apr 17;14:a28165093. doi: 10.1055/a-2816-5093 (PMC13294635; doi:10.1055/a-2816-5093)
Supplement: Supplementary file 1 — Supplementary Material [file 10-1055-a-2816-5093_28454912.pdf]

Supplementary Table 1 Summary of diagnostic criteria for JES classification.

Type A	Type A vessels have three or less of the following morphological factors: tortuosity, dilation, irregular caliber, and various shapes.<br>Type B vessels have all four of the following morphological factors: tortuosity, dilation, irregular caliber, and various shapes.
Type B1	Type B vessels with a loop-like formation
Type B2	Type B vessels without a loop-like formation
Type B3	Highly dilated vessels with calibers that appear to be more than three times that of usual B2 vessels

JES, Japan Esophageal Society.
